# A single amino acid substitution in SED-2 β-lactamase leads to increased inhibitor resistance and an extended substrate spectrum

**DOI:** 10.1128/spectrum.00224-25

**Published:** 2025-10-27

**Authors:** S. Laviad-Shitrit, H. Kon, D. Bychenko Banyas, R. Efrati Epchtien, M. Bechor, M. Lurie-Weinberger, E. Ben-Zeev, S. Frank, N. Rakovitsky, D. Schwartz, A. Keren-Paz, Y. Carmeli

**Affiliations:** 1 National Institute for Antibiotic Resistance and Infection Control, Israel Ministry of Health, Tel Aviv, Israel; 2 Medicinal Chemistry Unit, The Nancy and Stephen Grand Israel National Center for Personalized Medicine, Weizmann Institute of Science, Rehovot, Israel; 3 Faculty of Medical and Health Sciences, Tel Aviv University 26745, Tel Aviv, Israel; Seton Hall University, South Orange, New Jersey, USA

**Keywords:** extended spectrum beta-lactamase, beta-lactam resistance, inhibitor resistant, SED-2

## Abstract

β-Lactamases are the leading mechanism of resistance to β-lactam antibiotics in Enterobacterales. Here, we report the detection and characteristics of SED-2, a new member of the *Citrobacter sedlakii* class A chromosomal β-lactamase SED family. A *C. sedlakii* isolate recovered during routine screening was subjected to whole genome sequencing. A new variant of SED-1 was identified and named SED-2. *bla*SED-1 and *bla*SED-2 were cloned into *E. coli* DH10β. The donor *C. sedlakii* and the recombinant *Escherichia coli*-SED-1 and *E. coli*-SED-2 were further characterized. Antibiotic susceptibilities were determined using Sensititer. Kinetic assays were performed on purified SED-1 and SED-2. AlphaFold2 was used to predict the structure of SED-2 based on the primary amino acid sequence. *bla*SED-2 differed from *bla*SED-1 by six nucleotides, leading to one amino acid substitution (Q274R). Antibiotic susceptibility testing revealed that *bla*SED-1 and *bla*SED-2 conferred resistance to first-, second-, and third-generation cephalosporins but not to carbapenems. Kinetic studies of purified enzymes showed that SED-1 and SED-2 hydrolyzed ampicillin and cephalosporins but not meropenem. In comparison to SED-1, SED-2 had higher affinity and increased catalytic activity against the third-generation cephalosporins, especially ceftazidime. Moreover, compared with SED-1, SED-2 had a higher IC50 for clavulanic acid. 3D modeling of SED-2 predicted that the single amino acid substitution affected inhibitor-binding residues and led to a shift that destabilizes the enzyme active site, likely explaining the extended spectrum of activity. Our results highlight the diversity of β-lactamases naturally occurring in *Citrobacter* spp. We demonstrate here a rare example of a single amino acid substitution that simultaneously expanded the spectrum of β-lactamase activity by increasing affinity towards ceftazidime and reduced susceptibility to the β-lactamase inhibitor clavulanic acid.

**IMPORTANCE:**

In this work, we identified and characterized SED-2, a new member of the *Citrobacter sedlakii* class A chromosomal β-lactamase SED family. While only differing from the SED-1 by a single amino acid, the affinity of SED-2 towards the third-generation cephalosporin ceftazidime was dramatically increased. At the same time, its susceptibility to the β-lactamase inhibitor clavulanic acid was reduced. This is a rare example of a single amino acid substitution, occurring outside of the active site, simultaneously affecting two distinct enzyme functions.

## INTRODUCTION

Resistance to β-lactam antibiotics in Gram-negative bacteria is a serious global threat, with third-generation cephalosporin- and carbapenem-resistant Enterobacterales considered a critical group on the World Health Organization’s Bacterial Priority Pathogens List (WHO-BPPL) list in 2024 (1). The main mechanism of β-lactam resistance in Gram-negative bacteria is the production of β-lactamases, enzymes which hydrolyze the β-lactam ring, inactivating this class of antibiotics. Those ancient enzymes originally emerged in environmental bacteria to protect them from naturally occurring antibiotics. The widespread clinical use of β-lactams drove their rapid evolution and dissemination (2, 3). Point mutations changing key amino acids are known to contribute to β-lactamase evolution by extending substrate range and increasing catalytic efficiencies. One such case is the evolution of the Temoneira (TEM) and sulfhydryl variable (SHV) families from broad-spectrum to extended-spectrum β-lactamases (ESBLs) capable of hydrolyzing later-generation cephalosporins (4), while another is the evolution of the Guiana extended spectrum (GES) family ESBLs into carbapenemases (5). While the hallmark of ESBLs is that their hydrolysis activity is inhibited by β-lactamase inhibitors (such as clavulanic acid and tazobactam) (6), single amino acid substitutions have been shown to lead to an inhibitor-resistant (IR) phenotype (7, 8). SED-1 is a chromosomally encoded class A β-lactamase belonging to subgroup 2b in the Bush scheme. Initially described in *C. sedlakii* (9), it was later determined to represent a family of chromosomal class A β-lactamases sharing 84%–97% identity and present in four species: *C. sedlakii* (SED1), *C. amalonaticus* (SED1-like, formerly CdiA), *C. farmeri* (SED1-like), and *C. rodentium* (SED1-like) (10, 11). SED-1 confers resistance to aminopenicillins, carboxypenicillins, and first- and second-generation cephalosporins (e.g. cephalothin and cefuroxime). SED-1 has limited activity against some third-generation cephalosporins (such as cefotaxime), and no activity against acylureidopenicillins (such as piperacillin) or carbapenems (imipenem) (9). The crystal structure of SED-1 was described previously (https://www.rcsb.org/structure/3BFE). Here, we identify and characterize SED-2, a member of the SED-1 family, in which a single amino acid substitution resulted in the extension of the spectrum of substrates that it hydrolyzes efficiently and increased resistance to clavam inhibitors.

## MATERIALS AND METHODS

### Whole genome sequencing (WGS)

*C. sedlakii* 4972101 was isolated from a rectal swab collected for routine surveillance of carbapenem-resistant Enterobacterales conducted in a tertiary hospital in Israel in 2019. Initial ESBL activity was determined by a combination disc test (Oxoid Ltd, Basingstoke, UK), performed and interpreted according to Clinical and Laboratory Standards Institute (CLSI) M100 guidelines (12).

DNA was extracted using the MagAttract HMW DNA Kit (Qiagen, Hilden, Germany) and sequenced at Galil Genetic Analysis Ltd. using a Nextera XT library kit (Illumina Inc., CA, USA) on an Illumina Nextseq 500 device (2 × 150). Illumina reads were quality screened using Fastp (13), followed by *de novo* assembly using Spades (14). The resulting assembly was annotated with Prokka (15). Open reading frames (ORFs) were searched against the CARD and NCBI databases using Diamond Blast (16). The existence of *bla*SED-2 was verified using ResFinder 4.1 (https://genepi.food.dtu.dk/resfinderer/) (17). For amino acid comparison with other SED-1 enzymes, eight *C*. *sedlakii* strains with a complete genome available in the NCBI database were randomly chosen (ASM75983v1, ASM1650216v1, ASM1650785v1, ASM1812842v1, ASM3840537v1, ASM4041217v1, ASM4562534v1, and ASM4562606v1). ORFs were searched against the RAST database (https://rast.nmpdr.org/rast.cgi), and SED-1 amino acid sequences were aligned with MEGA7.0.26 (18) using the maximum likelihood method based on the Jones-Taylor-Thornton (JTT) matrix-based model.

### Cloning

Cloning was performed by amplifying both *bla*SED-1 and the suspected *bla*SED-2 gene using a primer set that included restriction sites: Forward (XbaI): CGACTCTAGAATGCTTAAAGAACGGTTTCG; Reverse (EcoRI): CAGTGAATTCTTACTTTCCTTCCGTCACAA. The amplified fragment was cleaned using the NucleoSpin Gel and PCR Clean-Up kit (Macherey-Nagel, Düren, Germany) according to the manufacturer’s instructions. The resulting PCR fragment was restricted by XbaI and EcoRI (New England Biolabs, Ipswich, MA, USA) and ligated into a pHSG396 plasmid with T4 DNA ligase (New England Biolabs). The resulting recombinant plasmids and an empty pHSG396 were transformed into *E. coli* DH10β competent cells (Invitrogen, Waltham, MA, USA). White colonies that grew on LB agar supplemented with chloramphenicol (25 µg/mL) and ChromoMax IPTG/X-Gal Solution (ThermoFisher Scientific, Waltham, MA, USA) were selected. The presence of the inserted *bla*SED-1 and *bla*SED-2 genes was confirmed by PCR followed by Sanger sequencing.

### Antibiotic susceptibility testing

Minimum inhibitory concentrations (MICs) of *C. sedlakii* 4972101, recombinant *E. coli*-SED-2 and *E. coli*-SED-1, and *E. coli* DH10β carrying an empty pHSG396 plasmid (negative control) were determined by broth microdilution (Sensititre DKMGN and ESBL EUVSED2 plates, ThermoFisher Scientific, Oakwood Village, OH, USA) and interpreted according to CLSI guidelines (19).

### Kinetic assays

Purified SED-1 and SED-2 (>85% purity by SDS-PAGE, produced by HIS-tagged expression in *E. coli* followed by affinity purification and tag removal) were purchased from GeneUniversal Inc. (Delaware, United States) and β-lactams were purchased from Sigma-Aldrich, Merck KGaA (Darmstadt, Germany). The kinetic parameters were determined by analyzing β-lactam hydrolysis under initial rate conditions in 50 mM phosphate buffer (pH 7.0), in a total volume of 200 µL, using 10 nM enzyme, at room temperature, using a Synergy HT microplate reader (BioTek Instruments, Winooski, VT, USA). The values of KM and kcat were calculated using the Michaelis-Menten equation and GraphPad nonlinear regression (GraphPad Prism 9). Enzyme inhibition was studied using ceftazidime (100 µM) as the substrate. The inhibitors were pre-incubated with each enzyme for 10 min at 25°C before adding the substrate. The inhibitor concentration required to inhibit 50% of the β-lactamase activity (IC50) was determined graphically for clavulanic acid and avibactam. We present the means of three independent measurements, each done in three technical repeats.

### Growth curves

Overnight cultures were adjusted to an optical density at 600 nm (OD600) of 0.001 in Luria-Bertani (LB) broth with and without antibiotics. The suspensions of each strain were inoculated in six replicates into a 96-well microplate (Greiner Bio-One, Kremsmünster, Austria). The cultures were incubated for 24 h at 37°C with shaking in a Synergy HT microplate reader (BioTek Instruments), and OD600 was measured every 30 min. The experiment was repeated three times, each time in three technical repeats.

### Protein modeling

AlphaFold2 (AF) (20, 21) was used to predict the structure of SED-2 based on the primary amino acid sequence. We selected the top-ranked Amber-based constrained-relaxation AlphaFold2 model for further structural analysis. The 3D structure model of SED-2 was visualized using Schrodinger’s Maestro tool. AlphaFold2 generated five top-ranked 3D atomic models after multiple sequence alignments and iterative structural assembly simulations based on the neural network. AlphaFold2 employed a final Amber-based constrained-relaxation step to remedy backbone and side-chain clashes. Model confidence and model accuracy were estimated by the AF confidence predicted local distance difference test (pLDDT) score and the predicted aligned error (PAE).

## RESULTS

### Gene identification

**Fig 1:**
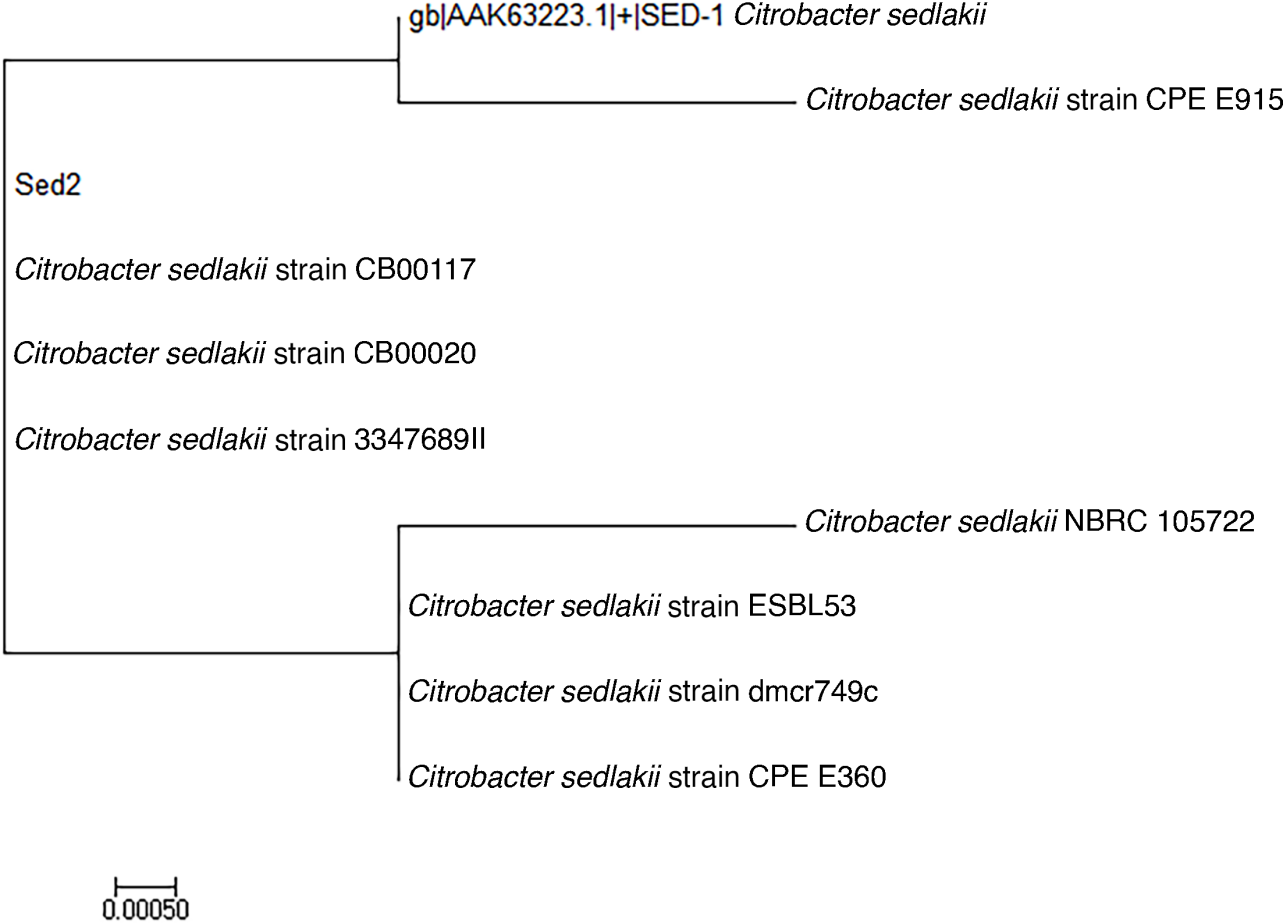
SED-1 family enzymes in *C. sedlakii*. Phylogenetic tree of amino acid sequences of SED-1. A tree with the highest log likelihood (−879.38) is shown. The scale bar represents substitutions per site.

*C. sedlakii* 4972101 had an ESBL phenotype by the combination disc test. WGS resulted in a 4,940,232 bp genome distributed into contigs, and 4,623 coding sequences (CDS) (including 75 tRNAs and 8 rRNA genes) were detected. No plasmids were detected. The genome contained full-length porin *ompA*, *ompD*, and *ompL* genes. Two β-lactamase genes were found: *ampH* and a homolog of *bla*SED-1. This homolog, which we designated *bla*SED-2, differed from *bla*SED-1 (accession number: AF321608) by six nucleotides: G9A, C177T, C330T, G624T, A762G, and A821G; five of these single nucleotide polymorphisms (SNPs) were silent. One SNP (A821G) led to a substitution of the uncharged glutamine residue by the positively charged arginine (Q274R). *bla*SED-2 was downstream to a gene encoding for a transcriptional regulator that was 99% identical to the SedR regulator previously described for *bla*SED-1 (accession number: AF321607), with two amino acid substitutions (S211N and M220L). To assess the frequency of the SED-2 variant in *C. sedlakii*, we randomly chose eight SED-1 family members from publicly available *C. sedlakii* genomic sequences. We found that 3/8 proteins were identical to SED-2 and different from SED-1 (Fig. 1). Moreover, arginine was present at position 274 in all sequences except for SED-1.

### Antibiotic susceptibility

**Fig 2:**
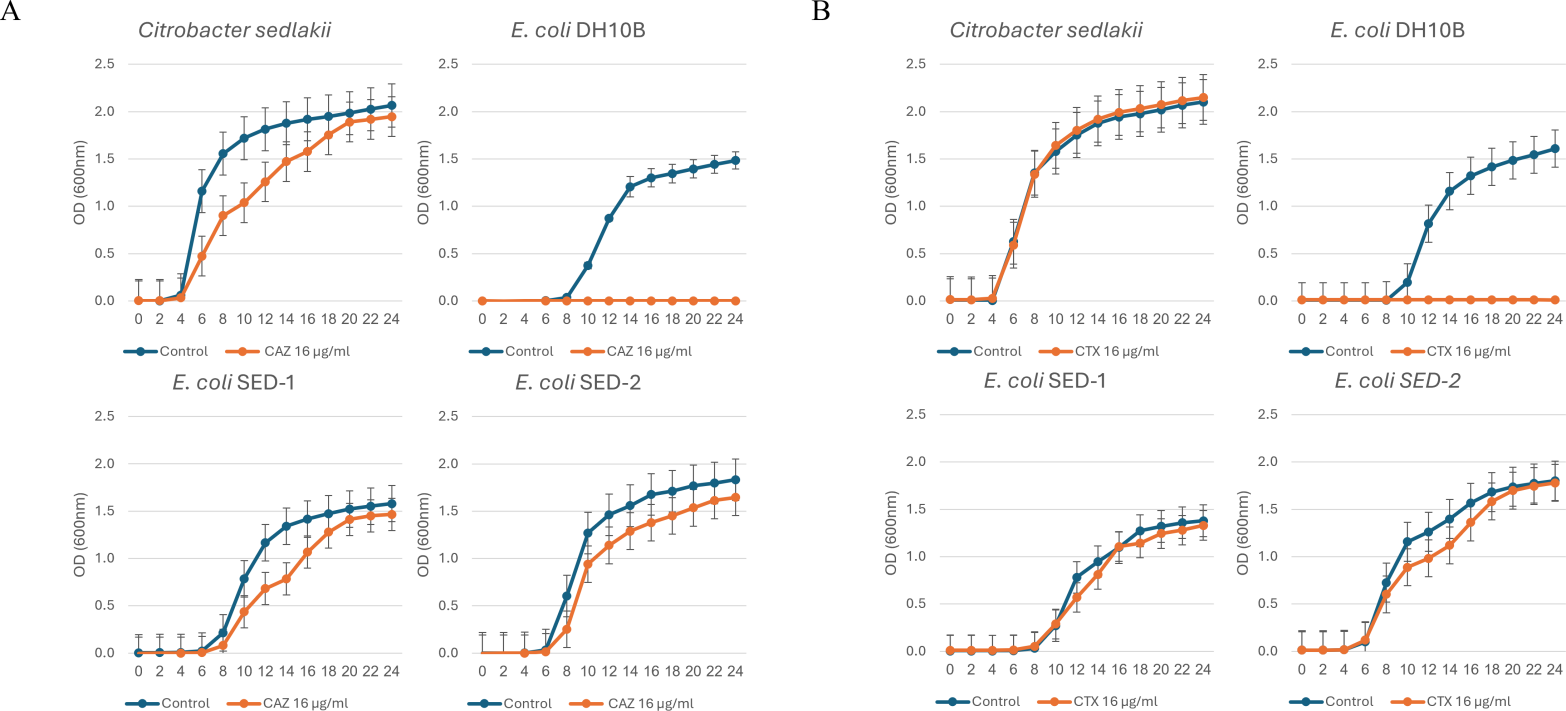
Growth curves. *C. sedlakii* 4972101, recombinant *E. coli-*SED-1 and *E. coli-*SED-2, and *E. coli* DH10β carrying an empty pHSG396 plasmid (negative control) were grown in BHI broth with and without: (**A**) ceftazidime (CAZ) (16 µg/mL), as indicated. (**B**) Cefotaxime (FOX) (16 µg/mL). Bars represent standard error.

To further characterize the activity of SED-1 and SED-2, we first expressed them in an *E. coli* DH10β strain. When grown in liquid medium, *C. sedlakii* 4972101 and recombinant *E. coli*-SED-1 and *E. coli*-SED-2 strains grew well on 16 µg/mL ceftazidime (CAZ) (Fig. 2A) and 16 µg/mL and 32 µg/mL cefotaxime (CTX) (Fig. 2B; Fig. S1), while the negative control did not. No significant difference was observed between the phenotypes of *E. coli*-SED-1 and *E. coli*-SED-2.

Broth microdilution revealed that the resistance profiles of *E. coli* expressing SED-1 and SED-2 were similar, and that both had an ESBL phenotype. SED-1/2 conferred resistance to penicillins, all cephalosporins tested (cefotaxime, ceftazidime, and cefepime), aztreonam, and ceftolazane/tazobactam but not to carbapenems and non-β-lactam antibiotics (Table 1).

**TABLE 1:** Antibiotic susceptibility profile^*a*^

	MIC (µg/mL)			
	*C. sedlakii* 4972101	*E. coli-*SED-2	*E. coli-*SED-1	*E. coli* (NC)
Amoxicillin/clavulanic acid	64/2	>64/2	>64/2	<4/2
Piperacillin/tazobactam	>32/4	>32/4	>32/4	2/4
Cefoxitin	8	8	8	1
Cefotaxime	16	32	32	>0.25
Cefotaxime/clavulanic acid	1/4	2/4	2/4	>0.06/4
Ceftazidime	16	16	16	≤0.25
Ceftazidime/clavulanic acid	2/4	4/4	4/4	0.25/4
Ceftazidime/avibactam	<05/4	≤0.5/4	≤0.5/4	<0.5/4
Cefepime	8	8	8	≤0.06
Ceftolozane/tazobactam	16/4	16/4	16/4	≤0.5/4
Aztreonam	≥32	≥32	≥32	≤0.5
Ertapenem	0.06	0.03	0.03	≤0.015
Imipenem	≤0.12	0.25	0.25	0.25
Meropenem	≤0.03	0.06	0.06	≤0.03
Ciprofloxacin	<0.06	<0.06	<0.06	<0.06
Amikacin	8	<4	<4	<4
Gentamicin	<0.5	<0.5	<0.5	<0.5
Trimethoprim/sulfamethoxazole	<1/19	<1/19	<1/19	<1/19

^*a*^ The MIC values for *C. sedlakii *4972101, recombinant *E. coli-*SED-1 and *E. coli-*SED-2, and the recipient *E. coli *carrying an empty pHSG396 plasmid (negative control, NC) were determined using broth microdilution (Sensititre DKMGN and ESBL EUVSED2 plates).

Table 2 shows the kinetic activity of SED-1 and SED-2. Both SED-1 and SED-2 had the highest catalytic efficiency against cephalothin. SED-2 had higher catalytic efficiencies against ampicillin and cephalosporins than SED-1 due to SED-2 displaying significantly lower Km values, especially for ceftazidime. Both SED-1 and SED-2 had low catalytic efficiency against meropenem. The IC50s determined using ceftazidime as a substrate showed that, compared with SED-1, SED-2 was inhibited less efficiently by clavulanic acid (22.2 µM for SED-2 vs 3.3 µM for SED-1). Taken together, these results demonstrate that the single amino acid substitution in SED-2 increased substrate affinity (especially towards ceftazidime) while simultaneously decreasing inhibition by clavulanate.

**TABLE 2:** Kinetic parameters of various β-lactam antibiotics for SED-1 and SED-2^*a*^

	SED-1				SED-2			
Substrate	Km (µM)	kcat (s^−1^)	kcat/Km (mM^−1^ s^−1^)	IC50 (µM)	Km (µM)	kcat (s^−1^)	kcat/Km (mM^−1^ s^−1^)	IC50 (µM)
Ampicillin	624.1 ± 94.0	30.9 ± 5.2	50.2 ± 7.9		308.0 ± 47.1	35.0 ± 5.8	107.4 ± 16.9	
Cephalothin	40.4 ± 6.5	12.9 ± 2.2	332.9 ± 35.2		36.0 ± 5.7	11.1 ± 2.3	307.1 ± 46.6	
Cefuroxime	50.9 ± 7.9	4.5 ± 0.6	88.8 ± 6.7		49.9 ± 7.9	8.8 ± 1.2	160.4 ± 23.9	
Cefotaxime	146.5 ± 21.1	15.1 ± 2.2	103.0 ± 15.5		82.2 ± 12.2	13.2 ± 2.0	157.9 ± 23.1	
Ceftazidime	1597.2 ± 235.7	62.5 ± 5.8	39.5 ± 5.4		63.3 ± 8.9	10.2 ± 1.4	159.0 ± 23.3	
Piperacillin	155.0 ± 15.3	2.9 ± 0.1	18.9 ± 2.6		41.3 ± 5.6	1.1 ± 0.1	24.5 ± 3.4	
Meropenem	206.5 ± 27.8	12.2 ± 1.5	59.1 ± 8.6		211.9 ± 20.5	11.4 ± 1.6	52.3 ± 7.7	
Clavulanic acid				3.3 ± 0.4				22.2 ± 2.8
Avibactam				7.9 ± 0.2				8.4 ± 0.1

^*a*^ Mean ± standard deviation values are indicated.

### Protein structure modeling

**Fig 3:**
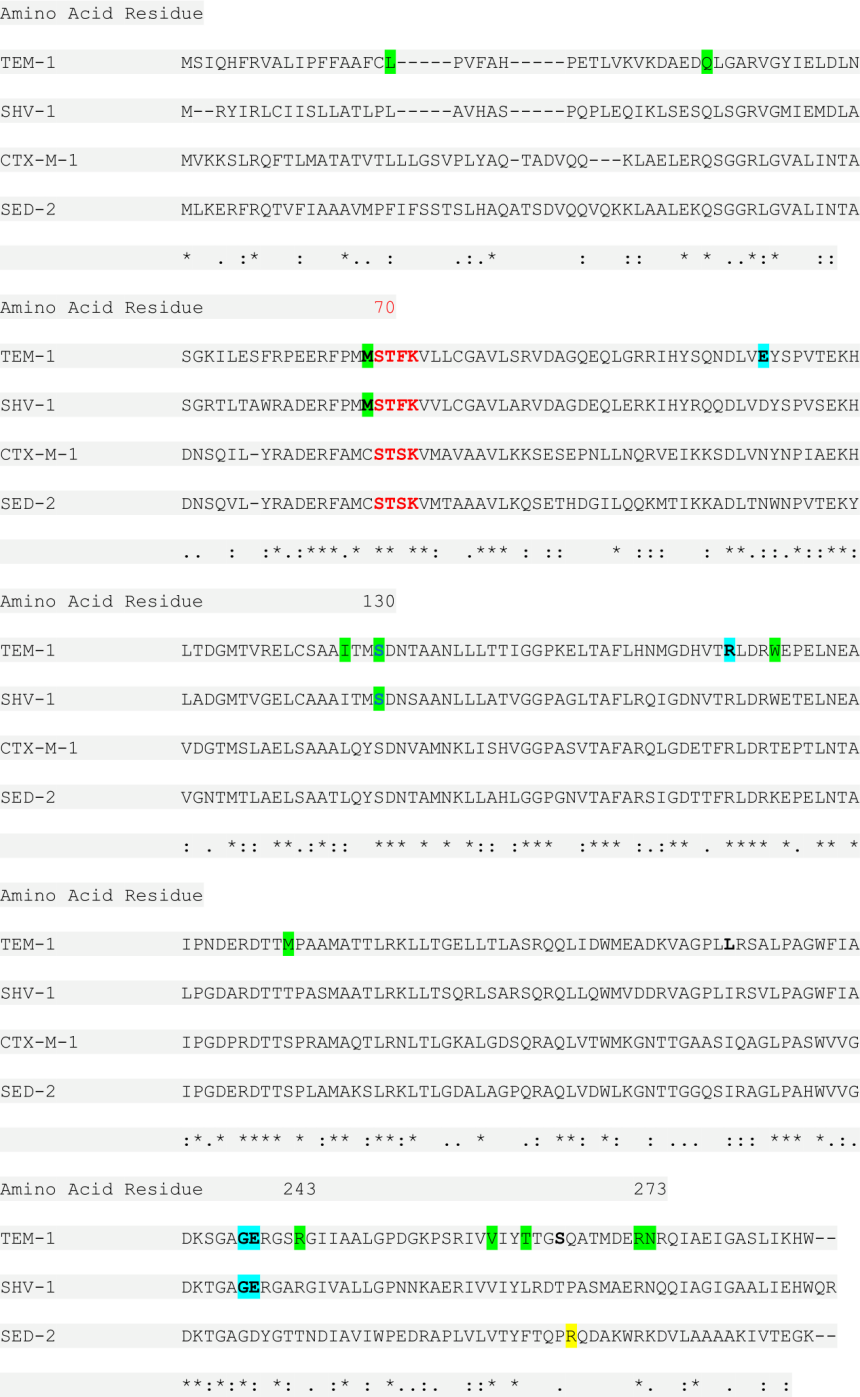
Amino acid alignments of TEM-1, SHV-1, CTX-M-1, and SED-2. Asterisk (*) indicates positions that have a fully conserved residue; Colon (:) indicates conservation between groups of strongly similar properties; Period (.) indicates conservation between groups of weakly similar properties. Red—the active site (Ser70-X-X-Lys) common to all serine β-lactamases, numbering as per the Ambler scheme position. Highlighted in blue—amino acid substitutions that confer an ESBL phenotype. Highlighted in green—amino acid substitutions that confer an IR phenotype. Highlighted in yellow—the substitution in SED-2.

**Fig 4:**
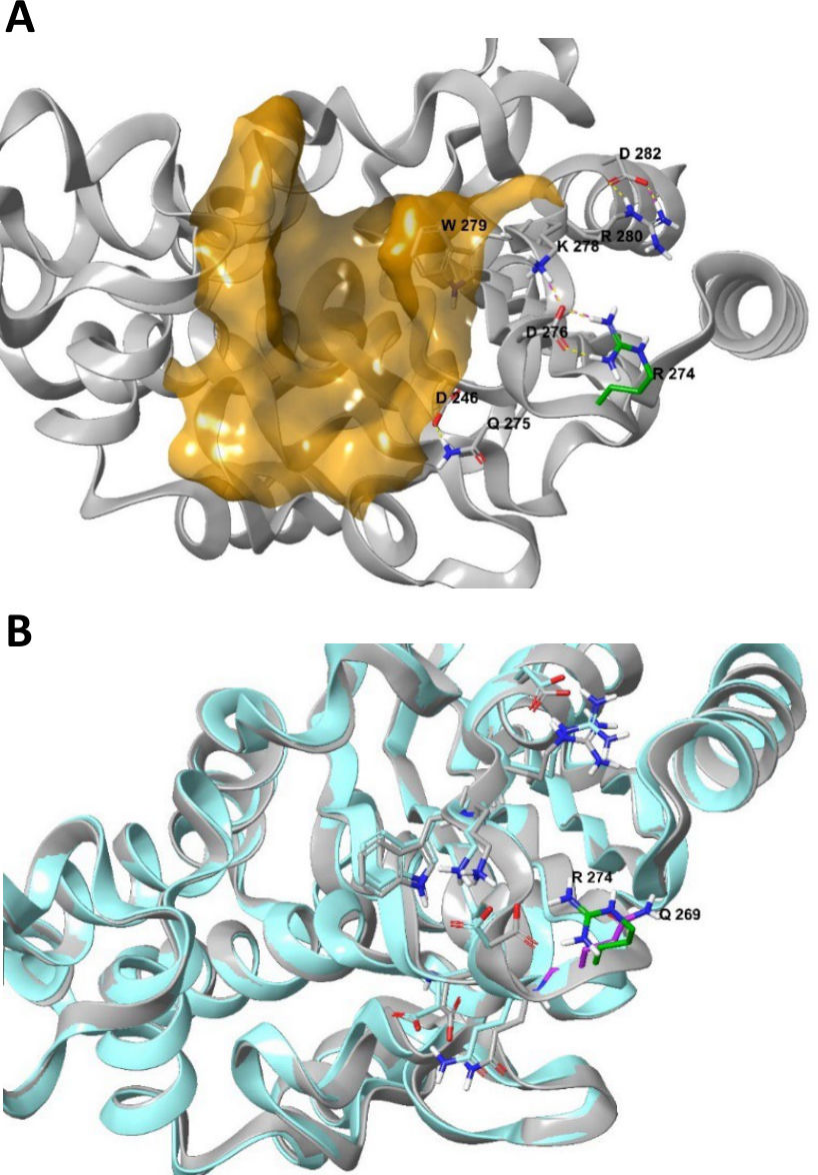
A model demonstrating the effect of a Q269 substitution on the SED-2 protein structure. (**A**) SED-2 AlphaFold 3D model: SED-2 is represented by the gray ribbon. The binding site is depicted as an orange surface. The amino acid residues are shown in a stick representation colored gray except for R274, which is in green. Salt-bridge and H-bond interactions between amino acid side chains are shown as purple and yellow dashed lines, respectively. (**B**) Superimposition of SED-2 on the SED-1 crystal structure. The colors of SED-2 are as described in panel **A**. SED-1 is represented by the blue ribbon. The amino acid residues are shown in a stick representation colored blue except for Q269, which is purple.

The equivalent of R274 in SED-2 is Q269 in TEM-1. While no activity-modifying substitutions have been reported at Q269 in the TEM structures, several mutations leading to an IR phenotype were reported in its vicinity (see Fig. 3) (22). Structural analysis of the SED-2 model revealed that, while the mutated R274 resides on a loop outside of the active site, it is capable of forming a contact network of acidic/basic amino acids in its vicinity: The D276 side chain interacts with K278 and R274 via a salt bridge or through H-bond interactions. D282 and R280 interact via H-bond and salt bridge, and D248 interacts with Q275 via H-bonds (Fig. 4A). Superimposing the SED-2 model and the SED-1 crystal structure (PDB: 3BFE, chain D; PDBDOI: https://doi.org/10.2210/pdb3BFE/pdb) shows that, in SED-2, the D276 network is missing, there is no interaction with Q269, the rest of the side chains adopt a different orientation and the protein backbone is slightly changed (Fig. 4B).

## DISCUSSION

We identified and characterized a new member of the SED-1 family, which we named SED-2, carrying a single amino acid substitution of uncharged glutamine to positively charged arginine (Q274R). Enzyme kinetics revealed that this single mutation led to an increase in catalytic activity against third-generation cephalosporins, especially ceftazidime, and to reduced inhibition by clavulanate. The increased activity was mostly due to higher affinity to the substrates, as reflected by the lower Km.

Single amino acid substitutions leading to IR or ESBL activity have been described for TEM and SHV (7, 8). Enzymes with a combination of several such mutations, leading to an IR ESBL phenotype, have also been described (6). However, to our knowledge, ours is the first report of a single mutation simultaneously extending substrate spectrum and increasing inhibitor resistance. The SED-1-Q to SED-2-R substitution inserts a positively charged residue into a network of salt-bridge and H-bond interactions, likely causing a change in the stabilization of the total structure. Analysis of superimposing SED-1 monomers from different crystal structures, as well as the different rotamer of SED-1-Trp (equivalent to SED-2-W279), shows that protein flexibility exists. This flexibility allows the ligands to bind in different modes. This is similar to the case of TEM-related ESBLs, in which mutations not in the active site may increase the plasticity of the active site, allowing its enlargement and leading to the ESBL phenotype (23). These findings support the hypothesis that the R274 substitution in SED-2 changes ligand and inhibitor affinity.

Despite differences in catalytic activity and inhibition by clavulanate, SED-2 and SED-1 conferred a similar resistance profile when expressed in the *E. coli* model system; resistance to amoxicillin, cephalothin, cefuroxime, cefotaxime, and ceftazidime, but not to meropenem. The discrepancy between the differences in the kinetic profile and the similar phenotype may be related to a high level of expression in the model system. In different stains, genetic backgrounds, or enzyme levels, differences between SED-1 and SED-2 kinetics may translate into phenotypic differences. We did not compare the expression levels of two enzymes in different strains, and this is a limitation of our study. This study underscores the diversity of naturally occurring β-lactamases and the potential for the emergence of novel enzymes, highlighting the importance of ongoing genomic surveillance.

## Data Availability

Data were submitted to NCBI under BioSample Accession Number PRJNA1012383 and Assembly Numbers GCA_031323735.1. The new SED-2 can be found under the accession number OR757576.179.

## SUPPLEMENTAL MATERIAL

Figure S1 spectrum.00224-25-s0001.docx Growth curves—32 µg/mL CTX.

ASM does not own the copyrights to Supplemental Material that may be linked to, or accessed through, an article. The authors have granted ASM a non-exclusive, world-wide license to publish the Supplemental Material files. Please contact the corresponding author directly for reuse.
