# Supplementary material for: A single amino acid substitution in SED-2 β-lactamase leads to increased inhibitor resistance and an extended substrate spectrum
Source: Microbiol Spectr. 2025 Oct 27;13(12):e00224-25. doi: 10.1128/spectrum.00224-25 (PMC12671189; doi:10.1128/spectrum.00224-25)
Supplement: Figure S1 — Growth curves—32 µg/mL CTX. [file spectrum.00224-25-s0001.docx]

**
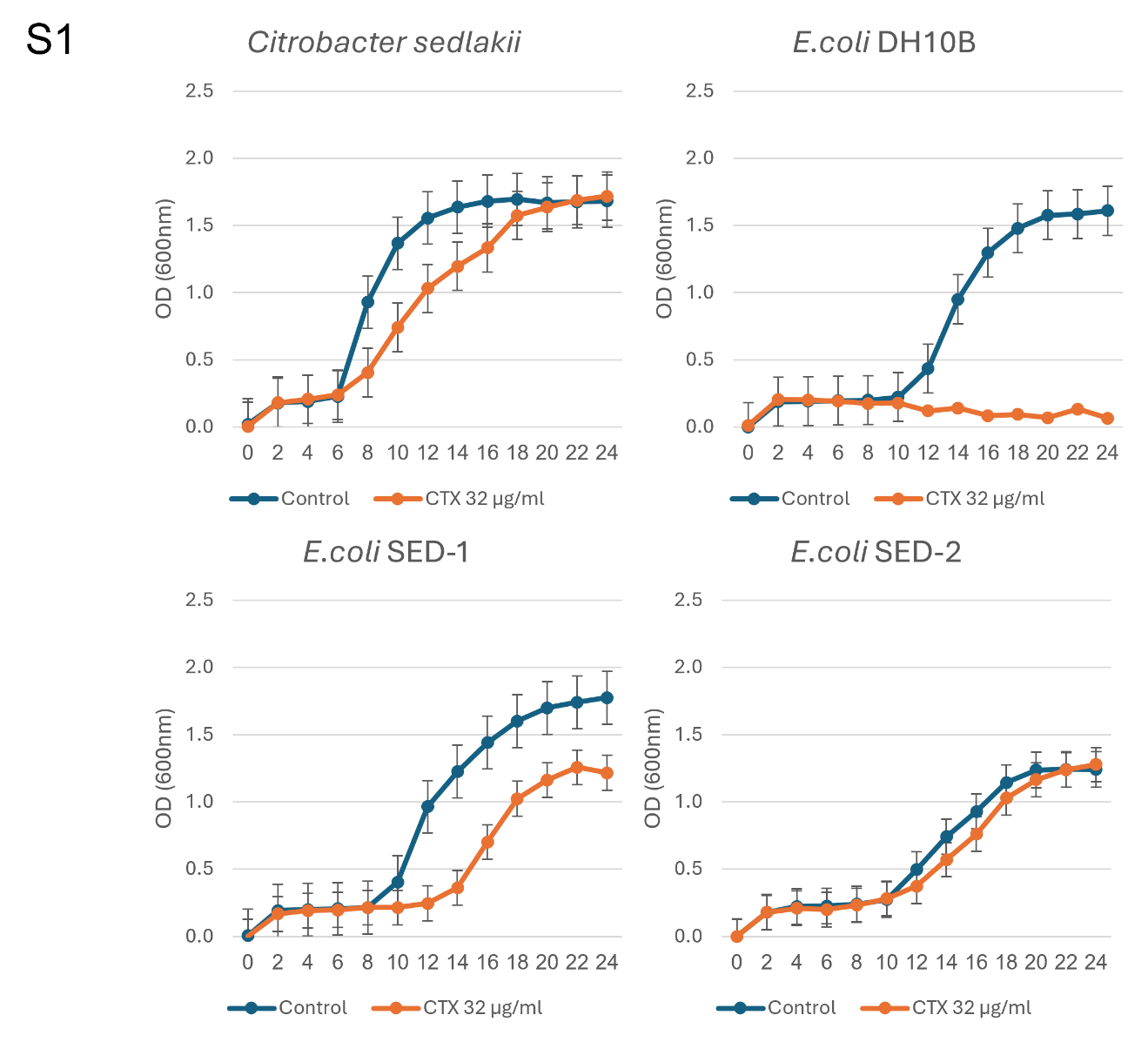
**

**Supplementary figure 1: Growth curves.**

*C. sedlakii* 4972101, recombinant *E. coli*-SED-1 and *E. coli*-SED-2, and *E. coli* DH10β carrying an empty pHSG396 plasmid (negative control) were grown in BHI broth with and without Cefotaxime (FOX), (32 µg/ml). Bars represent standard error.
